# London University College

**Published:** 1847-07

**Authors:** 


*London University College.*—The distribution of prizes in the medical department of this University took place on the 1st inst., and on the occasion the hall was filled with the students and their friends. Lord Brougham, as President of the University, ocupied the chair, and the proceedings opened with the reading of the annual report by Mr. Liston, Dean of the Faculty of Medicine. From that document it appeared that the number of medical students who attended classes during the past year was 290, the number of the preceding year being 292.—*Ibid.*

